# Radiogenomics of congenital brain malformations: Linking embryology, genetics, and imaging

**DOI:** 10.1007/s00234-026-03916-x

**Published:** 2026-02-11

**Authors:** Jehan AlRayahi, Khalid AlDasuqi, Marwa AlSubhi, Walid Mubarak, Osamah Al Walid

**Affiliations:** Department of Radiology, Sidra Medicine, Doha, Qatar

**Keywords:** Brain malformation, Embryology, Corticogenesis, Mosaicism, mTOR Pathway, Tubulin, Cytogenetic testing, Next generation sequencing

## Abstract

**Purpose:**

Congenital brain malformations are structural anomalies present at birth stemming from underlying genetic mutations or prenatal disruptions. The increased use of advanced genomic sequencing has led to major breakthroughs in pediatric neurogenetics; however, progress in unraveling the genetic basis of many central nervous system (CNS) malformations has lagged. This gap is partly due to the complexity of brain development and challenges like establishing the genetic culprit in somatic mosaicism. This review aims to integrate embryology, genetics, and neuroimaging to provide a practical radiogenomic framework for congenital brain malformations.

**Methods:**

A narrative review of the literature was performed focusing on fundamental embryologic processes of CNS development, genetic concepts relevant to malformations, and key molecular pathways and protein structures implicated in neurodevelopment. Representative malformations of cortical development, midline anomalies, and hindbrain malformations are discussed with emphasis on radiologic–genetic correlations.

**Results:**

Critical developmental pathways and proteins—including mTOR and Ras/MAPK signaling cascades and the tubulin cytoskeleton—are central to the pathogenesis of congenital brain malformations. Genetic principles, such as types of genetic alterations (e.g. somatic vs germline), mosaicism, penetrance, and expressivity explain the imaging and clinical phenotype variability and the diagnostic challenges encountered. Distinct radiogenomic patterns are identified across malformations of cortical development, corpus callosum anomalies, holoprosencephaly and posterior fossa malformations highlighting the diagnostic value of integrating neuroimaging with embryologic and molecular insights.

**Conclusion:**

Radiogenomic correlation of congenital brain malformations is increasingly important in the era of precision medicine. By correlating neuroimaging phenotypes with the relevant embryologic and molecular mechanism, neuroradiologists can improve diagnostic accuracy, guide genetic testing strategies, and contribute to multidisciplinary care and counseling.

## Key teaching points

The neuroradiologist’s role in brain malformation includes identifying likely gene culprits via pattern-recognition to guide genetic evaluation (e.g., selecting the appropriate gene panel). In the setting of an inconclusive genetic testing, the radiologist can assist by correlating variants of unknown significance with imaging features to assess pathogenicity—a process called “reverse phenotyping**”**.

Understanding stages of brain development helps appreciate pathogenesis of brain malformation. Failure of telencephalon division in week 5, for example, will result in holoprosencephaly. Errors in corticogenesis will result in different malformation of cortical development depending on the stage of insult. Defects in axonal guidance impair development of commissural fibers (e.g., corpus callosum) and projection fibers (e.g., corticospinal tract). Finally, errors in synaptogenesis may result in epilepsy with or without a clear structural malformation on imaging.

Mosaicism refers to the condition where different cells carry different genetic makeup within the same person. Its results from post-zygotic “somatic” genetic alterations. The pathogenicity of a somatic variant for brain malformation depends on 1) the type of mutational variant, 2) the type of progenitor cells affected and 3) the timing of the mutation.

The MAP Kinase/mTOR pathway is linked to countless tumors and vascular malformations. It is implicated in several malformations of cortical development including focal cortical dysplasia type IIB, tuberous sclerosis and other cortical malformations in the setting of overgrowth disorders.

The microtubule cytoskeleton is a dynamic structure crucial for proliferation, morphogenesis, migration, and cortical organization. Pathogenic variants in genes encoding for tubulin proteins (e.g. *TUBA1A)* or microtubule associated proteins (*LIS* or *DCX)* are linked with several brain malformations such as *tubulinopathies*andlissencephaly, respectively. Genes encoding other forms of cytoskeletal proteins key in corticogenesis are also implicated in brain malformation. Examples include those encoding for actin cytoskeleton proteins (e.g. *ACTB* &* ACTG1 *in lissencephaly) and cytoskeletal protein filamin A (e.g. *FLNA* in periventricular heterotopia).

## Introduction

Congenital malformations of the brain are structural abnormalities that develop in utero. They have a wide spectrum of clinical presentations, ranging from asymptomatic to seizures, hypotonia and developmental delay of variable severity. They can occur secondary to genetic and disruptive etiologies. The latter include prenatal infections as well as vascular and metabolic insults [1, 105]. 

Since the introduction of whole exome sequencing (WES) in 2005, there has been a plethora of breakthroughs in the genomics of pediatric central nervous system (CNS) disease, especially that of inherited neurometabolic diseases [3]. On the other hand, the pace of discovery in understanding the genetic pathophysiology of CNS malformations remains comparatively slow. To learn the reasoning behind this and the challenges met in the evaluation of CNS malformations, one must have a basic understanding of relevant embryology and genetics. This includes recognizing the different forms of genetic alterations encountered, the concept of genetic mosaicism, the available genetic testing techniques, and their limitations. One must also be familiar with key molecular pathways (e.g. mTOR pathway) and protein structures (e.g. tubulin) that govern the development of CNS malformations. In this paper, we will review these concepts and provide a brief overview of various CNS malformations and their genetic underpinnings.

## Genetic testing

Genetic testing includes the assessment of chromosomal structural abnormalities (i.e., cytogenetic testing) and more targeted analyses at the gene level. Cytogenetic testing encompasses (1) karyotyping which assesses numerical and large chromosomal abnormalities, and (2) chromosomal microarray analysis (CMA) which is more efficient and helpful in detecting smaller abnormalities such as copy-number variants (CNV) and single nucleotide polymorphisms (SNP). In CNVs, the number of specific DNA segments differs among individuals, whereas in SNP, variation occurs at a single nucleotide, the fundamental unit of DNA. More precise genetic testing has been divided into (1) targeted testing such as single gene or gene panel testing, and (2) non-targeted testing, such as whole exome or whole genome sequencing (WES and WGS, respectively) [1, 4]. When performed, even WES (or WGS) may be inconclusive with reports sometimes identifying “variants of unknown significance”. In such scenarios, genetic workup can be expanded to evaluate parents (also known as trio-WES) and affected family members, to better characterize the pathogenicity and inheritance pattern of these variants of unknown significance. A key limitation of this technique is encountered in conditions that demonstrate incomplete penetrance or variable expressivity. Penetrance is defined as the probability of the disease to manifest should one carry the pathogenic variant. Expressivity, on the other hand, represents the severity of disease presentation. A parent or relative might carry the pathogenic variant but remain unaffected because of incomplete penetrance or may exhibit a milder phenotype than the proband because of variable expressivity, making recognition of the culprit variant more difficult. Another method to assess inconclusive WES results is “reverse phenotyping”, where the clinician and radiologist re-assess the clinical and imaging phenotype of the disease, respectively, to find a correlation with the variants of unknown significance unveiled in WES or WGS. Finally, the quest for identifying the pathogenic variant may never halt; there are ongoing discoveries of novel genes and their phenotypic correlation, WES re-analysis and patient re-evaluation after a few years may be of benefit [5]. 

## CNS embryology

**Fig. 1:**
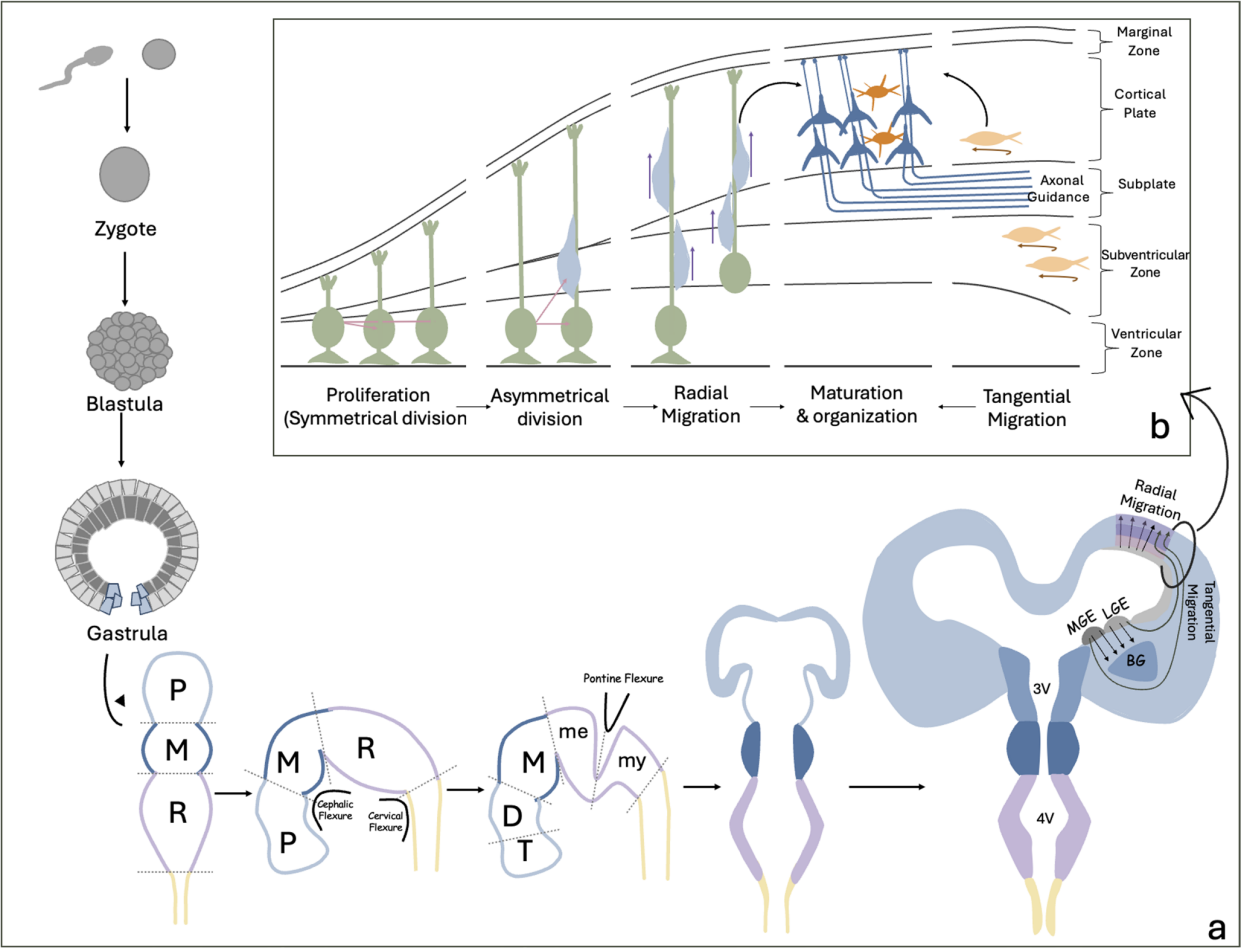
(**a**) Stages of Brain development. (**b**) Stages of cortical development. P: Porencephalon. M: Mesencephalon. R: Rhombo-encephalon. me: metencephalon. my: myelencephalon. 3 V: third ventricle. 4 V: fourth ventricle. MGE: Medial ganglionic eminence. LGE: Lateral ganglionic eminence. BG: Basal Ganglia

Understanding the stage-specific timeline of brain development during gestation is imperative to appreciate the heterogeneity of CNS malformations. Following fertilization and zygote formation, the zygote undergoes multiple divisions in a process called blastulation to form the blastula. By the third week, gastrulation occurs, giving rise to a trilaminar embryo composed of endoderm, mesoderm, and ectoderm—each destined to form specific tissue lineages [6]. The central nervous system evolves from the ectoderm through a process termed neurulation, in which the ectoderm thickens into the neural plate, folds inwards and closes to form the neural tube. The ventral part of the neural tube would encompass the basal plate responsible for the development of motor neuronal structures. The dorsal part of the neural tube would contain the alar plate, responsible for the development of sensory neurons and, later, the cerebellum. The neural tube transforms into three primary vesicles, the prosencephalon, the mesencephalon and the rhombencephalon, while the caudal end of the neural tube forms the spinal cord. The prosencephalon will expand with a high rate of apoptosis along its midline giving rise to a divided telencephalon and diencephalon that will eventually form the cerebrum and the thalami, respectively. Mesencephalon will, in due course, develop into the midbrain. The rhombencephalon will divide into the metencephalon and myelencephalon with the metencephalon eventually developing into the pons and cerebellum while the myelencephalon turns into the medulla. Concurrently, the neural tube bends at three flexures: the cephalic flexure (forebrain–brainstem angle), the cervical flexure (cervicomedullary junction), and the pontine flexure, which shapes the fourth ventricle. (Fig. 1a) [7].

**Fig. 2:**
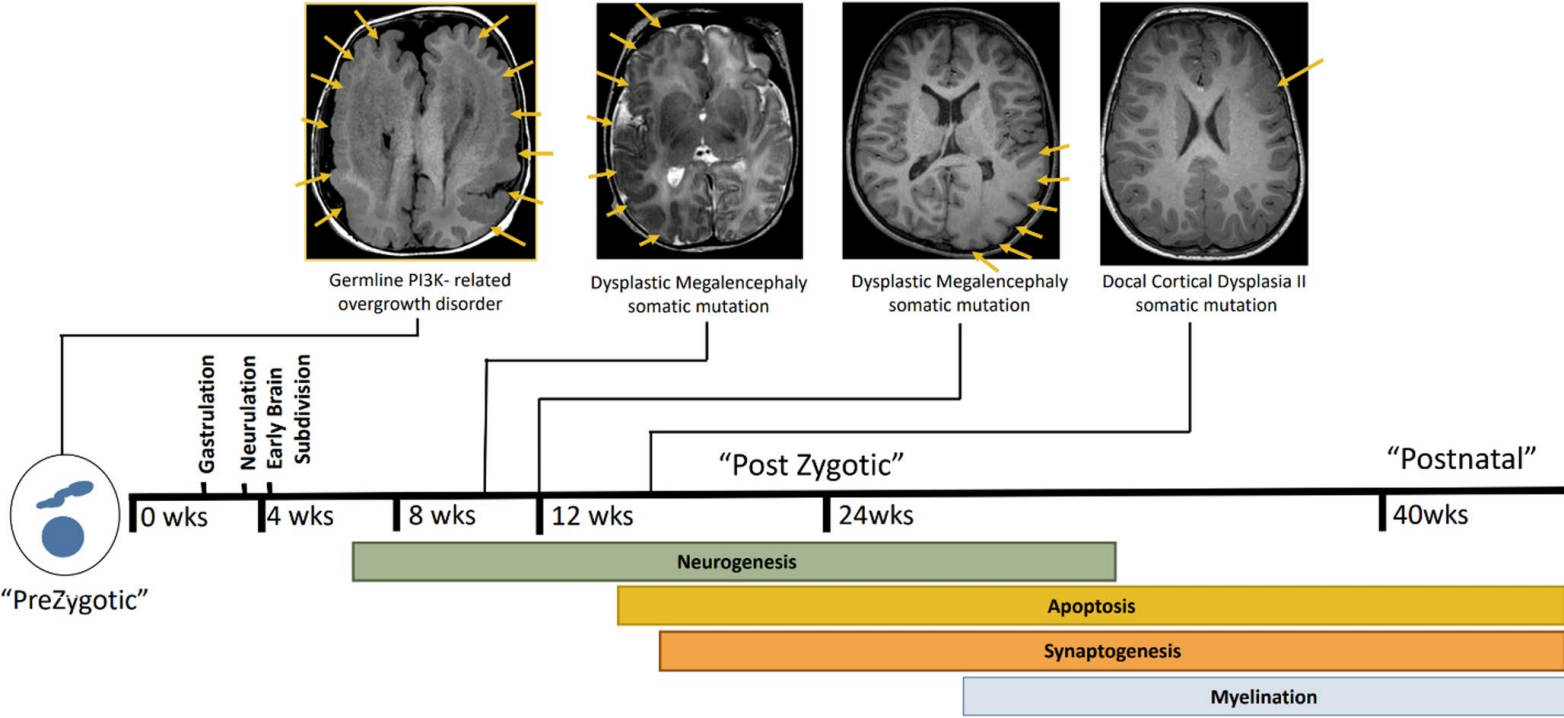
Timeline and stages of brain development

Upon the formation of brain vesicles, the more intricate development of grey and white matter will take place through several sequential and overlapping processes. This includes neurogenesis, apoptosis, synaptogenesis and myelination (Fig. 2). Neurogenesis begins at 4 weeks of gestation and is nearly complete by the third trimester. It represents the process during which progenitor cells undergo characteristic morphological and molecular changes to mature into neurons. During this stage, neuronal cells will also migrate to where they need to be (e.g., forming the cortex and deep grey matter) while their axons are guided to grow in a specific course to create white matter tracts and commissures in a process called axonal guidance or axonal pathfinding. Synaptogenesis (i.e., the process of “synapse” formation) begins at 12 weeks of gestation and helps in creating neuronal connections. It is later fine-tuned by synaptic pruning where excess dendrites and synapses are taken out. Apoptosis begins at 18 weeks, eliminating the overproduction of cells. Finally, myelination begins as early as 16 weeks of gestation and continues after birth [7, 8].

### Cerebral Hemispheres

Corticogenesis of the cerebral neocortex represents the formation of the 6-layer cortex. It takes place in three stages: (1) Proliferation, (2) Migration and (3) Organization (Fig. 1b). As the telencephalon develops, it is initially comprised of neuroepithelial cells spanning the full thickness of the neural tube with its apical end abutting the ventricular surface and the basal end contacting the pial surface. With maturation, the tube will configure into several transient laminar zones including the ventricular and subventricular zones that encompass the neuroepithelial progenitors, the cortical plate and overlying marginal zone which will later develop into the cortex, and finally the intermediate zone, which will become the future white matter, situated between subventricular zone and cortical plate. Within the ventricular zone, the neuroepithelial progenitors initially undergo rampant proliferation and later transform into radial glial cells that will continue to elongate as the parenchyma thickens, connecting the ventricles to the pial surface. The radial glial cells will also asymmetrically divide into (or indirectly form) neuronal precursors that will migrate into the cortex along the radial glial scaffold using it for guidance and as an anchor. This glial-guided “radial” mode of migration accounts for 90% of neuronal migration and is responsible for the migration of excitatory neurons into the cortical plateafter which they will detach from the radial glial cells and adjust their position within the cortex using another form of migration called “somal translocation”. In parallel, “tangential” migration is responsible for the migration of inhibitory neurons from the ganglionic eminence to the cortical plate via a longer route under complex regulatory control [7–10]. Finally, as neurons migrate into the cortical plate, their axons are projected into the intermediate zone to form the cortical white matter.

Corpus Callosum development takes place in multiple stages. It begins with the above mentioned neuronal migration and differentiation stages in corticogenesis, followed by the stages of axonal guidance prior to, and after, midline crossing and is finally concluded by the targeting stage where axons form synapses with the targeted neurons in the cortical plate of the contralateral hemisphere [11]. During the stage of axonal guidance, specialized dynamic structures, termed “growth cones”, found at the tip of these growing axons, take cues from the surrounding extracellular environment to navigate the axons to their target. The corpus callosum develops around the 8th week with the commissural fibers crossing the midline between 12 and 20 weeks. This process depends on the corpus callosum axonal growth cone receiving proper cues from molecules secreted and bound to the extra-axial structures to grow towards the midline. At the midline, several glial cell structures are formed including the “glial sling” and “glial wedge” which will produce cues to guide and support the axons across the midline. The first axons to cross the midline are those from the cingulate cortex which are termed “pioneering” axons. These pioneering axons also play a role in the guidance of following commissural axons. The development of the corpus callosum is believed to be bidirectional and the old theory that it forms in an anterior to posterior fashion has been negated. Initially the anterior commissural fibers arise in week 8, followed by the development of the hippocampal commissure by week 11. Early in week 13, the pioneering axons from the cingulate gyrus and anterior corpus callosum will begin to develop over the glial sling, while the rostral corpus callosum concomitantly crosses the midline over the hippocampal commissure. Further anterior commissural fiber development (associated with glial sling) will then fuse with the splenium of the corpus callosum (associated with the hippocampal commissure), thereby forming the entire corpus callosum. Given the rapid and greater extent of growth of the frontal lobes, the anterior corpus callosum will grow disproportionately to posterior segment, thereby displacing the hippocampal commissure and splenium posteriorly. Corpus callosum dysgenesis can be secondary to disruption of any of its stages of development including disruption of corticogenesis, midline patterning, axonal guidance and post guidance synaptogenesis [11, 12]. 

### Cerebellum

**Fig. 3:**
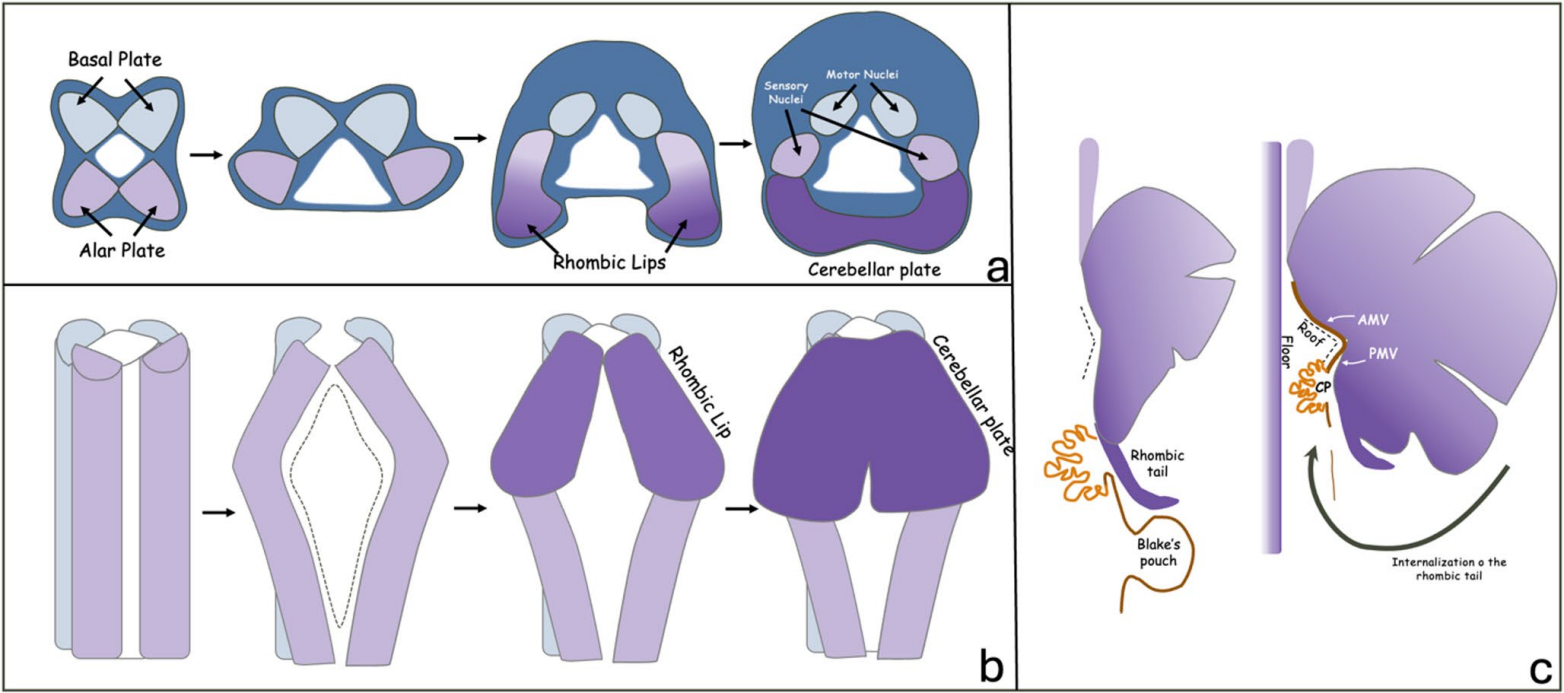
Development of the Cerebellum in axial (**a**), coronal (**b**) and sagittal (**c**) views. CP: Choroid plexus, AMV: Anterior medullary velum, PMV: Posterior medullary velum

With the formation of the pontine flexure, the alar plate starts to separate and the neural canal changes configuration to form the fourth ventricle which has a rhomboid-shaped floor ventrally and a roof dorsally. Two dorsal protrusions, defined as the rhombic lips, then arise from the lateral edges of the separated alar plate. These rhombic lips fuse along the midline in a craniocaudal direction, forming the cerebellar plate and eventually the cerebellum (Fig. 3) [13, 14]. The rhombic lip initially starts as a trigonal structure, giving rise to the cerebellum. As the cerebellum expands, the rhombic lips reconfigure into a tail-like structure at the caudal end of the developing cerebellum. At this stage, the fastigial point is flat. As the posterior cerebellum enlarges, however, the rhombic tail begins to be displaced anterosuperiorly, internalizing into the posterior-most lobule, thereby driving the fastigial point into an acute configuration [13, 15]. 

During the tenth week of gestation, the developing choroid plexus indents the roof of the fourth ventricle, dividing it into the superior medullary velum (or anterior membranous area) and the inferior medullary velum (or posterior membranous area). The inferior medullary velum subsequently fills with CSF to form Blake’s pouch cyst, which in most cases later perforates, creating the foramen of Magendie from as early as 10 weeks and usually before 24–25 gestational weeks. The cerebellum develops at the 5th week of gestation and continues to differentiate until the age of two years [13]. 

## Genetic mosaicism and its challenges

Congenital brain malformations may result from genetic alterations in human DNA prior to, or after, the formation of the zygote. Germline alterations take place “pre-zygotic” only and can be present in one or both parents. Accordingly, all cells of the embryo formed will carry the germline mutation. Somatic alterations, on the other hand, are not present in either parent, but transpire in the “post-zygotic” stage.

**Fig. 4:**
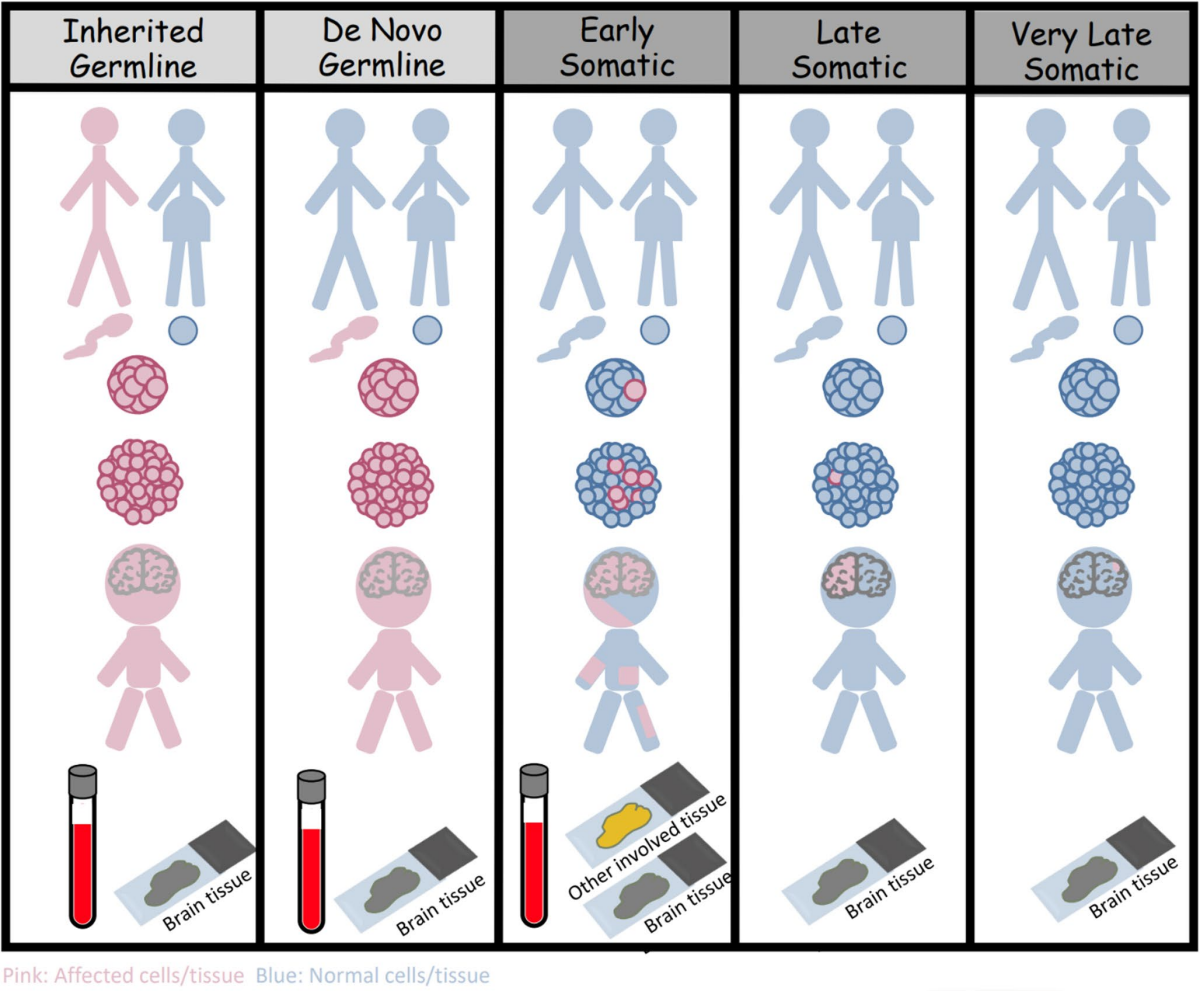
Effect of Mutation type and timing on subsequent clinical phenotype and tissue involvement. Pink: Affected cells/tissue. Blue: Normal cells/tissue

Somatic mutation can occur anytime from conception to death. Most somatic variants accumulate in utero when neuronal cell division is at its peak and takes place at a much slower rate postnatally. Over 90% of somatic variants are SNVs, with a smaller percentage being CNVs and only rarely aneuploidies. It is essential to be aware that normal brain cells harbor hundreds of somatic variants. This complicates genetic evaluation, as variants that are not clearly pathogenic are often reported as “variants of unknown significance”. The pathogenicity of a somatic variant for brain malformation depends on (1) the type of mutational variant, (2) the type of progenitor cells affected and (3) the timing of the mutation [6, 16]. As discussed, there is a high number of variants that can exist in normal cells, highlighting that not every variant has clinical consequences. The type of progenitor cells affected is also key because a mutation that may drive a neuronal progenitor into developing a malformation may not do so in other cell lineages (e.g., hematopoietic progenitor) [16]. Finally, the timing of the DNA alteration significantly influences the outcome. If it occurs early during embryogenesis, for example, prior to gastrulation, then the abnormality will be found in most cell types, including blood. If the somatic alteration takes place early after gastrulation, then it may involve neuronal progenitors only and the resultant DNA abnormality may only be detected in CNS tissue, but not in blood. Finally, somatic alterations occurring during later stages of cell division will affect “committed” differentiated neuronal cell progenitor, and will be restricted to the cell lineage of that specific progenitor and not all CNS tissue and surely not in blood (Figs. 2 and 4)[6, 9, 16]. Understandably, somatic alteration that occur prior to hemispheric division will result in bilateral brain malformations.

**Fig. 5:**
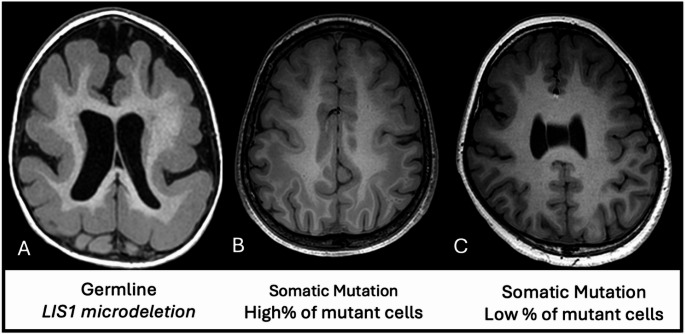
Effect of variant allelic fraction (VAF) in lissencephaly-pachygyria spectrum severity phenotype. Select T1 images of different patients with lissencephaly-pachygyria spectrum malformation, showing higher phenotype severity when the variant is germline (VAF of 50%) (**A**) in comparison to patients with somatic variants and lower VAF values (**B **& **C**)

Mosaicism constitutes the biggest challenge in understanding the genetic pathophysiology of congenital brain malformations because the genetic abnormality may not be detectable in blood and may be present only in the affected brain tissue, which can be analyzed only if surgically resected. Moreover, even when brain tissue is available, genetic analysis may be difficult because it depends on the quality of genetic testing performed, and more importantly, the genetic disease burden of the specimen. The frequency at which a pathogenic variant can be detected in a specimen is known as “variant allelic fraction (VAF)”, a metric that depends on the number of abnormal gene/cells relative to normal ones in the specimen. Tissues with germline mutation, for example, would have a VAF of 50% if inherited from one parent, as only half of the genome is affected, and 100% if inherited from both parents. Somatic mutation resulting in hyperproliferative malformation such as hemimegalencephaly occurs earlier during cell division and so can have a high VAF of approximately 30%, whereas, lesions resulting from somatic mutations during later stages of cell division, such as focal cortical dysplasia, will affect a small part of the brain and will also have a low VAF of approximately 5%. This is because the specimen often contains a small number of abnormal cells, diluted by surrounding non-mutated cells (Fig 0.2,4)[6, 9, 16, 17]. VAF not only correlates with the size of the abnormality, but in certain malformations, the severity of phenotype as well. A good example is that of the lissencephaly-pachygyria spectrum. Germline mutations of *DCX*, an X-linked gene, would result in 100% VAF in male patients as it is considered “hemizygous” and 50% VAF in females where the variant is “heterozygous”. Accordingly, male patients will have a more severe phenotype of lissencephaly whereas female patients will present with double cortex. If the *DCX* variant is somatic, then it will show a less severe phenotype (Fig. 5).

## Malformation of cortical development

Malformation of cortical development accounts for nearly 40% of refractory epilepsy[12]. They are classified into disorders secondary to defects in proliferation or apoptosis (e.g., microcephaly), disorders secondary to disruption of migration (e.g., heterotopia, lissencephaly and cobblestone dysplasia) and finally disorders secondary to inappropriate post-migrational cellular organization (e.g., focal cortical dysplasia type I)[10, 14]. This is an oversimplification as several malformations such as tuberous sclerosis or focal cortical dysplasia type II, may involve multiple stages of cortical development.

###  The MAP kinase pathway & related cortical malformations

**Fig. 6:**
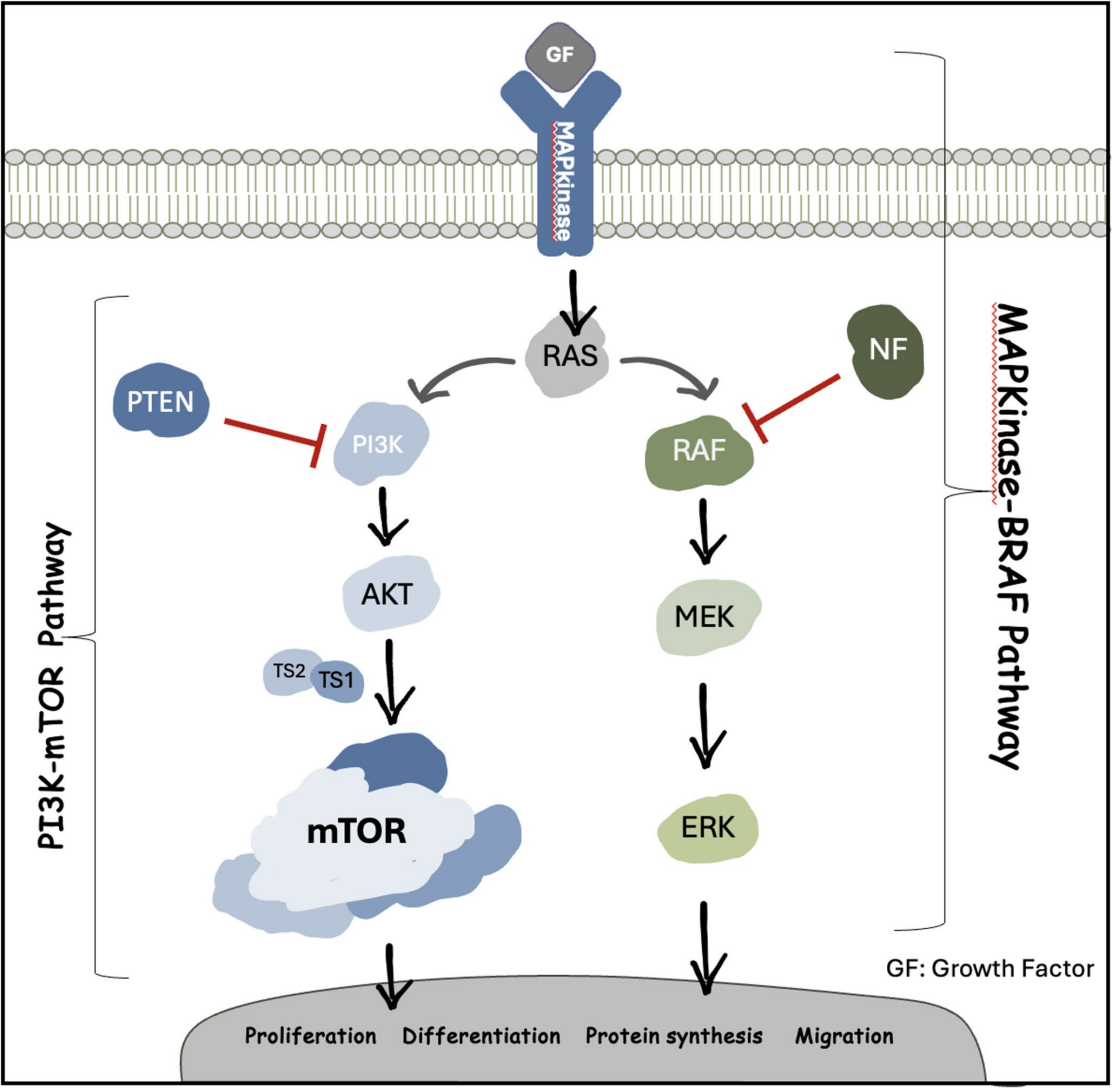
MAP Kinase pathway

The MAP kinase pathway is one of the very few pathways that a pediatric neuroradiologist may be required to be familiar with. It is one of the most frequently involved pathways in tumors, vascular malformations and malformations of cortical development (Table 1). It is initiated by a growth factor binding to the transmembrane receptor tyrosine kinase which in turn transiently activates a cascade of reactions that will ultimately govern DNA transcription and translation into proteins that promote cell division, migration, differentiation and survival. The MAP kinase pathway starts with the activation of the RAS protein which then activates two downstream pathways, the mTOR pathway and the BRAF-MAPK pathway. The mTOR pathway involves PI3K, AKT, GATOR1 Complex, RHEB, mTOR, TSC1 and TSC2 proteins and the BRAF-MAPK involves BRAF, MEK, and ERK proteins (Fig. 6) [18, 19]. GATOR1 complex, a negative regulator of mTOR, is not a common culprit of FCD II, though it is noteworthy as the genes encoding its components *(DEPDC5*, *NPRL2* and *NPRL3*) are major contributors of “non-lesional” focal refractory epilepsies and in some cases of Rolandic epilepsy and infantile spasm [20–22].

**Table 1:** Malformation of cortical development

Malformation of Cortical Development	Notes	Implicated genetic abnormalities
FCD I	FCD IA: Related to alteration in pathway of Glycosylation FCD IB & IC: Unknown etiology – May have a disruptive etiology at post-migrational stages	*SLC35A2*,* CANT1* (in FCD 1a)
FCDII	Alteration in mTOR pathway	*mTOR > TSC1*,* TSC2 > PIK3CA*,* PTEN*,* DEPDC5*
FCD III	Etiology unknown. Alterations in BRAF-MAPK pathway have been implicated. May have a disruptive etiology at post-migrational stages.	*BRAF*,* FGFR2*,* NOD2*,* and MAPK9*
Tuberous sclerosis	Alteration in mTOR pathway	*TSC1*,* TSC2*
Hemimegalencephaly	Alteration in mTOR pathway	*PIK3CA*,* PTEN > mTOR > TSC1/2*
Overgrowth disorders	Alteration in mTOR pathway affecting multiple tissues Mutation timing and type of cell involved determines severity and extent of disease	*PIK3CD*,* PIK3R1/2*,* mTOR*,* AKT3*,* PTEN*,* TSC1/2*,* AKT*,* DEPDC5*
Tubulinopathy	The tubulin protein forms the microtubule cytoskeleton which has numerous functions in cell support, cell division, morphogenesis, migration, axonal guidance amongst many more.	*TUBA1A*,* TUBA8*,* TUBB2B*,* TUBB3*,* TUBB5 and TUBG1*
Lissencephaly and lissencephaly-Pachygyria spectrum	Lissencephaly and lissencephaly-Pachygyria spectrum are often linked to genes related to tubulin, microtubule- associated proteins and other extracellular proteins	**MAPS**: *LIS1*,* DCX* *CDK5*,* DYNC1H1*,* KIF2A*,* KIF5C*,* NDE1/NDEL1*, **Tubulin genes**: *TUBA1A*,* TUBA8*,* TUBB*,* TUBB2B*,* TUBB3*,* TUBG1* **Others**: *ARX*,* RLN*,* VLDLR*, *CRADD*,* ACTB*,* ACTG1*,
Cobblestone cortex	Cobblestone cortex *without* muscle and eye involvement includes Merosin deficient congenital muscular dystrophy. Cobblestone cortex *with* muscle and eye involvement results from dysfunction of the alpha-dystroglycan protein. Radiological associations include white matter disease and various posterior fossa anomalies including dysmorphic cerebellum with subcortical cysts. Cobblestone cortex *with* muscle and eye involvement include: 1) Fukuyama congenital muscular dystrophy which has a temporooccipital distribution 2) Muscle-eye-brain syndrome. 3) Walker-Warburg Syndrome is most severe and lethal. Associated with hydrocephalus and kinked brainstem appearance. *ADGRG1*/*GPR56* related brain disease show “cobblestone-like” polymicrogyria, white matter abnormalities, and cerebellar hypoplasia	*LAMA2* (in merosin deficient congenital muscular dystrophy) *POMT1*,* POMT2*,* and FKRP* (in cobblestone cortex with muscle and eye involvement) *ADGRG1/GPR56* (in “cobblestone-like” polymicrogyria)
Polymicrogyria	Approximately two thirds have disruptive etiologies such as antenatal ischemic insult and infection (e.g., CMV) Genetic etiologies include hundreds of genes and multiple chromosomal abnormalities. Common monogenic etiologies were linked to tubulinopathies, PI3K-m-TOR pathway and less commonly collagenopathy (e.g., COLA4A1/A2). The most common chromosomal abnormality is 22q11.2 deletion, which encompasses DiGeorge syndrome	Chromosomal: 22q11.2 *TUB genes*,* PIK3R2*,* GPR56*, *WDR62*,* COL4A1/A2 COL18A1*,* RTTN*,* SRPX2*,* PAX6*,* EOMES*,* KIAA1279*,
Heterotopia	X-linked heterotopia linked to *FLNA* is most common and demonstrates a posterior predominance periventricular heterotopia, possibly associated with corpus callosum anomalies, cerebellar hypoplasia and mega cisterna magna. It has strong systemic associations such as cardiovascular anomalies and connective tissue disorders warranting workup. When focal or unilateral, heterotopia is likely secondary to prenatal disruptive etiologies and may not warrant genetic evaluation unless patient is syndromic or have a strong family history.	*FLNA*,* ARGFGEF2>* *NEDD4L*,* MAP1B*,* DCHS1*,* FAT4*,* EML*,* GPSM2* 6q27 deletion

**Fig. 7:**
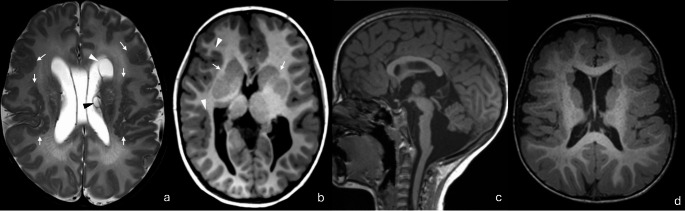
(**a**) Polymicrogyria in *PI3K*-related overgrowth disorder*.* Bilateral perisylvian and frontal polymicrogyria (arrows) are associated with megalencephaly and germinolytic cysts (arrowheads). Differential diagnoses for this appearance include Zellweger syndrome (**b**, **c**) Tubulinopathy*.* Note the dysmorphic basal ganglia, with associated dysgenesis or agenesis of the anterior limb of the internal capsule (B, arrows), and scattered areas of polymicrogyria (B, arrowheads), in addition to the dysmorphic ventricles, abnormally shortened corpus callosum, dysplastic brainstem, and dysgenetic and hypoplastic vermis (C) (d) *NEDD4L*-related malformation. Bilateral periventricular nodular heterotopia and bilateral frontal and perisylvian polymicrogyria and dysgyria

The mTOR pathway promotes cell growth, proliferation and survival and has been implicated in tuberous sclerosis, focal cortical dysplasia type II (FCDII), hemimegalencephaly and overgrowth disorders [6, 10, 23]. Tuberous sclerosis specifically requires a “two-hit” loss of function mutation of *TSC1*/*TSC2* genes, one germline and inherited from the parents, and the second takes place as a somatic variant. These genes encode the tuberous sclerosis proteins which are tumor suppressors that act as negative regulators of the mTOR pathway. FCD II is defined as cortical dyslamination with dysmorphic neurons, with (FCD IIa) or without (FCD IIb) balloon cells. FCD II is known to harbor somatic variants in *mTOR*, *and less frequently TSC1 and TSC2*, with a smaller percentage of lesions harboring *PIK3CA*, *PTEN* and *DEPDC5* variants. Hemimegalencephaly, on the other hand, primarily carries *PIK3CA*, *PTEN* and *mTOR* variants with a smaller number carrying *TSC*, *AKT1* and *DEPDC5* variants [6]. Finally, many overgrowth disorders such as megalencephaly-capillary malformation syndrome, CLOVES (congenital lipomatous overgrowth, vascular malformations, epidermal nevi, scoliosis syndrome) and Proteus syndrome are termed PIK3CA-related overgrowth syndromes (Fig. 7a) [23]. In this group of disorders, mosaicism plays a major role in presentation as the timing of somatic mutation and the progenitor affected will determine the severity of changes and the body parts involved. Accordingly, analysis of affected tissue (e.g., cheek swab or organ biopsy) is more useful than blood (Fig. 4). FCD III is defined as cortical dyslamination taking place in conjunction with another lesion such as hippocampal sclerosis in FCD IIIa, tumor in FCD IIIb, vascular malformation in FCD IIIc, and gliosis in FCD IIId. The etiology of FCD III remains unknown, though there are studies linking it to the BRAF-MAPK pathway. This finding may explain the presence of concomitant lesions in FCD types IIIb and IIIc because the BRAF-MAPK pathway is also implicated in most gliomas as well as vascular malformations [18, 19, 24]. FCD IIId is thought to result from a prenatal or early postnatal acquired insult [25, 26].

Finally, FCD type I, described as radial and/or tangential dyslamination, as well as mild malformation of cortical development with oligodendroglial hyperplasia (MOGHE), have not been linked to the MAP Kinase pathway. They remain an enigma, with only FCD Ia, which features radial lamination, and MOGHE found to be related to somatic mutations of *SLC35A2* and *CANT1* glycosylation genes [17]. FCD Ib, characterized by tangential dyslamination and FCD Ic, characterized by both radial and tangential dyslamination, however, have not been genetically determined [27]. One theory proposes that these may not represent true malformations but rather the sequelae of prenatal disruptive insults [28]. 

### Tubulin and related brain malformation

The tubulin protein consists of two subunits, α-tubulin and β-tubulin, which together form the building block of microtubules, a hollow and polar tubular structure that makes up the largest filaments of the neuronal cytoskeleton [29]. Microtubules interact with microtubule-associated proteins & motors (MAPs) which regulate their stability and assembly. The microtubule cytoskeleton has a unique feature termed “dynamic instability” which allows the microtubules to alternate between phases of “polymerization” (elongation) and “depolymerization” (shortening). Accordingly, the microtubule cytoskeleton can rapidly remodel to perform diverse roles during brain development. This includes contributing to processes such as proliferation, migration, maturation, axonal guidance and cellular support. Microtubules, for example, aid in proliferation by creating the mitotic spindles at opposite poles of the cell during mitosis. These mitotic spindles constantly change length, attaching to chromosomes, promoting their alignment and then shortening to exert force and divide the chromosomes. During migration, the tubulin cytoskeleton forms a cage-like structure around the nucleus, generating forces that move it toward its target. During axonal guidance, microtubules provide structural support for axons and dendrite growth and maturation, and facilitate intracellular transport along the axon [29, 30]. 

Tubulinopathies are inherited neurological disorders secondary to mutations in the tubulin gene family (*TUBA1A*, *TUBA8*, *TUBB2B*, *TUBB3*, *TUBB5* and *TUBG1*) [30]. They have a vast range of radiological phenotypes, not limited to CNS malformation, but also include hypomyelinating disorders (e.g. hypomyelination with atrophy of the basal ganglia and cerebellum (H-ABC)) and degenerative diseases (e.g. progressive spastic paraplegia and congenital fibrosis of extraocular muscles (CFEOM))[32, 33]. The constellation of findings often described in tubulinopathies include cortical malformation, corpus callosum dysgenesis and posterior fossa abnormalities. In addition, fused basal ganglia has been described as a hallmark of tubulinopathies (Fig. 7b) [30]. These abnormalities can be explained by the known function of tubulin. Given its role in migration, tubulin mutation may result in under-migration or over-migration and dyslamination may result in lissencephaly or polymicrogyria, respectively. On the other hand, because tubulin plays a role in axonal guidance, then related mutations may result in dysfunction in the formation of the commissural fibers of the corpus callosum, or the white matter tracts that form the anterior limb of the internal capsule which would result in the fused appearance of the basal ganglia. Similarly, disruption of axonal growth of the corticofugal tracts into the brainstem may contribute to brainstem hypoplasia [29, 33]. Interestingly, asymmetry of these malformations (e.g., asymmetrical hypoplasia of the pons) is considered a diagnostic clue for tubulinopathy. Finally, it is important to note that this is an oversimplification of the many functions of microtubules with extensive research being performed in attempts to understand the regulatory mechanism of tubulin.

### Other malformation of cortical development

#### Lissencephaly

**Fig. 8:**
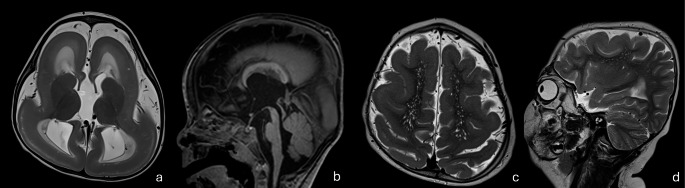
(**a**, **b**) Miller-Dieker syndrome with de novo deletion in 17p13 linked to* LIS.* Diffuse lissencephaly showing markedly thickened cortex, smooth cerebral outlined, with absent cortical sulci and shallow sylvian fissures, giving the figure of “8” appearance. The corpus callosum is dysgenetic and hypoplastic anteriorly (**c**, **d**) Anterior predominance Lissencephaly and prominent perivascular spaces in patient with *ACTB1 *mutation

Microtubule- associated proteins (MAPs) are proteins that regulate tubulin function and stabilization. They include structural MAPs such as *LIS1* and *DCX* and motor proteins such as Kinesins and Dynein [29]. Interestingly, a few of the earliest genes implicated in disorders of cortical malformation are those coding for the MAPs LIS1 (aka PAFAH1B1) and DCX which were initially described in 1993 and 1998, respectively [24, 34]. The LIS1 protein interacts with microtubules during migration and axonal guidance, whereas *DCX* is expressed during proliferation, differentiation and migration. Mutations in *LIS1* and *DCX* are often germline but can be somatic and are linked to Lissencephaly-pachygyria spectrum of disorders. *LIS1* is also linked to Miller-Dieker syndrome (Fig. 8a, b), which is associated with microcephaly, facial dysmorphism and demonstrate poor prognosis, with most affected patients not surviving beyond early childhood [35, 36]. Since *DCX* is X-linked, it usually results in lissencephaly in male patients and double cortex in female individuals (Fig. 4) [29]. *LIS1* typically results in a posterior to anterior severity gradient (i.e. phenotype appears more sever posteriorly), while *DCX* results in an anterior to posterior gradient [37]. Genes encoding the motor protein Kinesin and Dynein have also been implicated in lissencephaly, though much less commonly. These include *KIF2A*, *KIF5C* [38–40] from the kinesin superfamily genes and *DYNC1H1* gene from the Dynein gene family, the latter better known for its link to Charcot-Marie-Tooth disease [29, 41, 42] and spinal muscular atrophy [29, 42, 43].

Other than the tubulin cytoskeleton, the extracellular matrix also plays a crucial role in corticogenesis by providing cues for neuronal migration, morphogenesis and cortical organization. The *RELN* gene which encodes for an extracellular protein responsible for guidance during neuronal migration is implicated in lissencephaly, as is the gene encoding *VLDLR*, a RELN receptor protein. Reelin pathway related genes are also highly expressed in the cerebellum and so, the phenotype is often associated with severe cerebellar hypoplasia[45–47]. Other pertinent genes related to lissencephaly include *NDE1*,* NDEL1*,* CDK5*,* ARX* [47], *CRADD* [48] and actin genes (*ACTB & ACTG1*) [37, 49]. NudE homolog proteins (NDE1, NDEL1) aid in the regulation of LIS1 and Dyenin, and are linked to lissencephaly, microcephaly, as well as hypoplasia of the corpus callosum, brainstem and cerebellum. Similarly, variants in CDK5, a regulator of DCX and LIS, can cause severe and at times lethal lissencephaly as well as corpus callosum dysgenesis and severe cerebellar hypoplasia. *ARX* is an X-linked gene that plays a role in proliferation, differentiation and specifically tangential migration, and has been described in X-linked LIS with abnormal genitalia. Understandably, male patients have a more severe phenotype and present with abnormal testicular and pancreatic function as well as concomitant corpus callosum dysgenesis and striatal and thalamic atrophy on MRI [47]. *CRADD* plays an anti-apoptotic role, and loss-of-function variants have been described in mild thin lissencephaly, megalencephaly and intellectual disability [48]. *ACTB* & *ACTG1* genes enocde for proteins that are part of the actin cytoskeleton, another cytoskeleton that plays a role in neuronal migration. It often presents with an anterior-predominant pachygyria, prominent perivascular spaces with an absent or thickened corpus callosum and relatively normal posterior fossa (Fig. 8c, d). Baraitser–Winter malformation syndrome is a syndromic association in which patients also present with posterior heterotopia, facial dysmorphism and intellectual disability [37, 49]. Radiogenomic correlations in lissencephaly depends on overall severity of phenotype, anatomical gradient of severity and associated anomalies. The cortical thickness of lissencephaly vary from 10 to 20 mm with those measuring 5–10 mm, categorized at “thin” lissencephaly [50]. In severe phenotypes, such as agyria, candidate genes should include *DCX*, *LIS1*, *TUBB*-family genes, and *CDK5*. Asymmetric basal ganglia dysmorphology/incomplete separation in conjunction with asymmetric pontine and cerebellar hyopoplasia/dysgenesis suggests a tubulinopathy whereas severe lissencephaly, marked cerebellar hypopoplasia, and corpus callosum dysgenesis could be related to either a tubulinopathy or an underlying CDK5 gene variant. Anterior predominance in pachygyria/agyria spectrum phenotype suggests *DCX*, *ACTB*/*ACTG1*, *CRADD* and *RELN*/*VLDLR*, the latter especially if associated with severe cerebellar hypoplasia. Posterior predominance in pachygyria/agyria spectrum phenotype suggests *LIS1* and *ARX *involvement. *ARX* specifically should be considered when associated with deep grey matter atrophy and/or in the setting of endocrine dysfunction, a condition known as X-linked lissencephaly with abnormal genitalia (XLAG). Finally, thin lissencephaly with associated megalencephaly would hint at *CRADD* as the pathogenic variant [37]. 

#### Cobblestone cortex

**Fig. 9:**
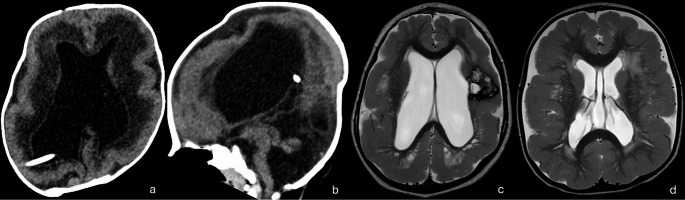
(**a**, **b**) Cobblestone Cortex in Walker Warburg Syndrome. Diffuse cobblestone cortex, shunted hydrocephalus with dysmorphic ventricles, and absent septum pellucidum on axial CT (A). Z-shaped configuration of the brainstem and hypoplastic vermis on sagittal reconstruction (B) (**c**) Cobblestone Cortex in *LAMA2 *mutation*. *Unrelated left periventricular hemorrhagic infarct present (**d**) Cobblestone-like polymicrogyria in* GPR56 *mutation

Cobblestone cortex can be divided into those with associated muscle and/or eye disease and those without. The most common diseases associated with cobblestone cortex with muscle and eye involvement include Fukuyama congenital muscular dystrophy, muscle-eye-brain syndrome, and Walker-Warburg syndrome (Fig. 9a, b), all of which are often associated with white matter, brainstem, and cerebellar abnormalities. They develop as a result of dysfunction of the α-dystroglycan protein which is responsible for the formation and stabilization of the glia limitans, a layer of astrocyte foot-processes covering the cortex underneath the pial membrane. Disruption of the glia limitans will in turn result in gaps in the layer and allow neuronal over-migration through these gaps into the subarachnoid space giving the cobblestone appearance [51]. These syndromes have several radiogenomic correlations. Fukuyama congenital muscular dystrophy is due to *FKTN* gene mutation and shows a temporooccipital prevalence of cobblestone malformation. Posterior fossa anomalies include fused colliculi, and dysmorphic cerebellum with subcortical cysts. Muscle-eye-brain syndrome is linked to several mutations including that of *POMGNT1* gene. It often presents with diffuse white matter abnormalities, midline anomalies such as corpus callosum dysgenesis and absent septum pellucidum, as well as posterior fossa anomalies including midbrain tectal plate fusion, pontocerebellar hypoplasia and cerebellar dysplasia with subcortical cysts. Walker-Warburg syndrome carries the most severe phenotype and is lethal. Its genetic etiology is not well-known; however, it has been linked to several genes including *POMT1*, *POMT2*, and *FKRP* genes. Walker-Warburg syndrome demonstrates prominent and extensive cobblestone phenotype, with a classical “kinked” appearance of the mesencephalic-pontine junction and hydrocephalus as well as various other posterior fossa anomalies including pontocerebellar hypoplasia, cerebellar dysplasia with cysts and encephalocele formation [52–56].

An example of cobblestone cortex without muscle and eye involvement is merosin-deficient congenital muscular dystrophy, which results from variant in the *LAMA2* gene (Fig. 9c). The merosin protein is also important for the integrity of the glia limitans layer. Merosin deficiency leads to defects in the glia limitans layer, allowing for neuronal over-migration and the cobblestone appearance[58]. Interestingly, the clinical and radiological spectrum of Laminin-related muscular dystrophy is very wide, with milder forms showing delayed motor development, whereas the severe form can present with severe hypotonia and a weak cry from birth. Several series have described cortical malformations to present in only 10–40% of cases [58]. Cobblestone cortex, when present, shows a posterior predominance. Associated findings include white matter abnormalities with variable patterns of distribution and severity, a deep interpeduncular cistern, cerebellar hypoplasia and cerebellar cysts [59–61]. 

Finally, mutations in *GPR56/ADGRG1* gene result in pial interruption leading to over-migration and bilateral frontoparietal “cobblestone-like” polymicrogyria, white matter abnormalities, as well as cerebellar hypoplasia (Fig. 9d). There is ongoing debate as to whether *GPR56* should be classified within the true cobblestone malformations of cortical development or within the “cobblestone-like” polymicrogyria spectrum [62].

**Fig. 10:**
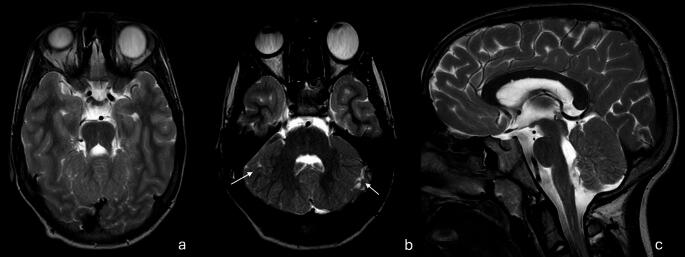
(**a**-**c**) Poretti-Boltshauser syndrome (*LAMA1* mutation)*.* Abnormal posterior fossa with molar tooth-like midbrain (a), dysplastic cerebellum with subcortical cysts (arrows). Left globe deformity also noted

Given the association of cobblestone cortex with posterior fossa anomalies and cerebellar subcortical cysts, Poretti-Boltshauser syndrome, a neuro-ophthalmological disorder related to *LAMA1* mutation is often considered in the differential (Fig. 10). Poretti-Boltshauser syndrome can present with pontine and cerebellar hypoplasia, brainstem malformation (including a molar tooth-like sign) and ocular findings, similar to known associations of several congenital muscular dystrophies as well as Joubert disease.

#### Polymicrogyria

Polymicrogyria results from disruption of late neuronal migration and cortical organization. It may be focal, diffuse, symmetrical or asymmetrical and usually categorized based on its distribution. Perisylvian polymicrogyria is the most common form (60–70%) followed by generalized polymicrogyria [63], polymicrogyria with periventricular grey matter heterotopia (such as the example of Baraitser–Winter malformation syndrome, discussed earlier) and frontal polymicrogyria. A substantial proportion of polymicrogyria is caused by prenatal disruptive injuries such as antenatal ischemic insult or infection (e.g., CMV and Zika virus) and has been linked to maternal drug abuse [64]. Accordingly, accurate history and neuroimaging features of an insult, such as temporal cysts and calcification in congenital CMV, are important. Genetically, it is linked to hundreds of genes including tubulin genes (e.g., *TUBA1A*, *TUBB2B*, *TUBB3*) (Fig. 7b, c) and genes related to mTOR pathway (e.g., *PIK3R2* or *PIK3CA*) (Fig. 7a). Collagenopathies (e.g., *COL4A1* and *COL4A2)* are also implicated in polymicrogyria. Accordingly, WES/WGS is the preferred method of genetic evaluation, in patients with a positive family history or in cases where neuroimaging findings cannot be explained by disruptions. Finally, chromosomal abnormalities include chromosome 22q11 deletion syndrome, various chromosomal rearrangement syndromes and chromosomal aneuploidies (e.g., Trisomy 13 and Turner mosaicism). 22q11.2 deletion encompasses DiGeorge syndrome which is also known as velocardiofacial syndrome. As the name implies, patients often present with facial dysmorphism, cleft palate and cardiac anomalies. Other noteworthy syndromes associated with polymicrogyria are Zellweger, Aicardi and Walker-Warburg syndrome [65]. 

#### Heterotopia

Heterotopia represents constituently normal but ectopic grey matter found along the route of a neuronal migration disturbance. It includes periventricular heterotopia, subcortical heterotopia and mixed periventricular and subcortical heterotopia. Periventricular heterotopia may have bilateral symmetrical distribution, bilateral asymmetrical distribution, unilateral distribution or be focal. Subcortical heterotopia may be diffuse, multilobar, unilateral or focal.

Periventricular heterotopia is the most common form, with over 60 gene associations, the most common being *FLNA*, followed by *ARFGEF2* [14, 66]. The *FLNA* gene specifically encodes for cytoskeletal protein filamin A, responsible for the scaffolding for neuronal migration from the ventricular to the subventricular zones. *FLNA* is X-linked and so primarily presents in female patients since pathogenic variants are typically lethal in hemizygous males. *FLNA* variants demonstrate a posterior predominance periventricular heterotopia and are associated with corpus callosum anomalies, cerebellar hypoplasia and mega cisterna magna and present with mild cognitive disability and seizure. More importantly, they have strong systemic association with cardiac anomalies, vasculopathy and stroke, highlighting the importance of diagnosis for early management and counseling [67]. Periventricular heterotopia linked to *ARFGEF2* variants affect male and female patients equally and show a distinct presentation including microcephaly, severe intellectual disability, spastic quadriplegia and possibly cardiomyopathy [68–70]. When associated with other brain malformations including cortical malformation such as polymicrogyria, and corpus callosum anomalies, then *NEDD4L* [66, 71, 72] (Fig. 7d), *MAP1B* [66, 73], and occasionally *DCHS1/FAT4* variants should be considered [65, 74]. *DCHS1/FAT4* variants specifically are linked to Van Maldergem syndrome where periventricular heterotopia may be nodular or rather confluent and smooth in appearance, termed laminar heterotopia. This syndrome is associate with distinct facial features, intellectual disability as well as ear and skeletal anomalies [66, 74, 75]. Finally, periventricular heterotopia – whether unilateral, focal, or bilateral – may be associated with prenatal disruptive etiologies such as ischemia, hemorrhage, or congenital infection (e.g., CMV), and imaging studies should be examined for supportive features such as associated encephalomalacia. These disruptive processes are thought to interfere with neuronal attachment to and migration along radial glial fibers. Importantly, bilateral periventricular heterotopia does not exclude a disruptive etiology and is frequently encountered in this setting. However, marked asymmetry, unilaterality, or focality may provide stronger imaging support for a prenatal disruptive process. In such cases, genetic evaluation may be of lower yield in the absence of syndromic features or a relevant family history.

Multilobar subcortical heterotopia are linked to *EML1*,* DCHS1 and FAT4* variants, especially when associated with cobblestone-like cortex and ventriculomegaly [66]. *EML1 * specifically is linked to ribbon-like heterotopia syndrome, characterized by megalencephaly, “ribbon -like” extensive contiguous subcortical heterotopia, polymicrogyria and corpus callosum agenesis. Patients with this syndrome present with refractory seizures, significant intellectual disability and ophthalmological abnormalities [76]. A rare but noteworthy syndrome is Chudley McCullough syndrome related to *GPSM2* mutation where the subcortical heterotopia may appear “comma- shaped” or in contiguous clusters parallel to the ventricles. Associations include corpus callosum dysgenesis, ventriculomegaly, interhemispheric cyst and cerebellar dysplasia. These patients present with hearing loss, seizures and intellectual disability [77, 78]. The combination of multilobar subcortical heterotopia and periventricular heterotopia suggests *MAP1B*,* DCHS1*,* FAT4* variants and at times *FLNA* mosaicism, the latter suggesting consideration of mosaic testing if WES is inconclusive. Similar to focal periventricular heterotopia, focal subcortical heterotopia is often secondary to prenatal disruptive etiologies, most commonly due to a vascular insult.

Finally, heterotopia has also been associated with several numerical and structural chromosomal abnormalities including CNVs. There is an extensive list of single gene and chromosomal abnormalities linked to heterotopia, many with syndromic association. Gene panels are available and used if the clinical or imaging suggests a specific etiology. Testing, otherwise, relies on both cytogenetic and WES/WGS analysis [1, 66]. 

## Corpus callosum anomalies

Corpus callosum abnormalities are genetically heterogeneous, described at least once in approximately 400 rare diseases and linked to over 360 genes or chromosomal loci [79]. Agenesis of the corpus callosum may be complete or partial, reflecting arrest in axonal crossing during embryogenesis. Hypoplasia refers to a structurally intact but diffusely thinned callosum, usually due to reduced axonal numbers or abnormal myelination [80]. “Dysgenesis” is a controversial term that has sometimes been applied to partial agenesis or abnormal morphology (akin to dysplasia in other classifications [81]). Secondary destruction describes acquired callosal loss or thinning following insults such as hypoxic–ischemic injury, infection, or severe hydrocephalus. Less commonly, a thickened corpus callosum may be observed in overgrowth disorders (e.g., *PIK3CA*-related overgrowth spectrum), neurofibromatosis 1, autism spectrum disorder, schizophrenia, or even in the normal population [82]. Presentation can range from asymptomatic to severe neurodevelopmental disorder and even death. It is one of the most common brain malformations with an incidence of 0.5–7.5 per 10,000 births [83]. More importantly, it is one of the most common brain anomalies diagnosed prenatally with ultrasound which warrants prenatal counseling. Prenatal counseling is influenced by the results of the genetic work-up which can detect an abnormality in approximately 55% of cases. It is also influenced by the presence or absence of associated anomalies (both CNS and extra-CNS). This is because the presence of associated anomalies has been shown to predict a worse prognosis, and fetal MRI may then be warranted. Overall, approximately half of corpus callosum anomalies present in isolated form, 23% are syndromic and 25% occur with other anomalies [84, 85]. Prenatal workup includes karyotype, CMA and possibly WES. Karyotype and CMA abnormalities can be detected in approximately 10% of cases while monogenic etiologies can be identified by WES in up to 35% of fetuses [85]. 

## Holoprosencephaly

**Fig. 11:**
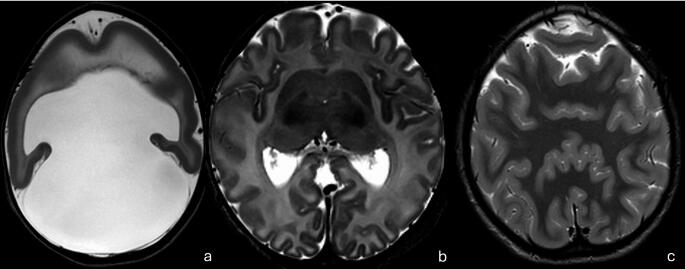
(**a**) Alobar Holoprosencephaly. Single midline monoventricle, with abnormal midline structures (absent septum pellucidum, agenesis of corpus callosum, absent interhemispheric fissure and falx cerebri) (**b**) Semilobar Holoprosencephaly. Complete separation of the cerebral hemispheres posteriorly. Absent falx cerebri anteriorly, agenesis of corpus callosum, partly fused frontal lobes, fused basal ganglia and thalami (**c**) Middle Interhemispheric Variant (Syntelencephaly). Fused posterior frontal and anterior parietal lobes at the vertex

Holoprosencephaly develops when the prosencephalon (i.e., forebrain) fails to form two separate lobes during the third and fourth weeks of gestation. Its spectrum of presentation includes various degrees of fusion and includes alobar holoprosencephaly, semi-lobar holoprosencephaly and lobar holoprosencephaly. Alobar holoprosencephaly is the most severe form, with a pancake appearance of the residual fused parenchyma and the dorsal mono-ventricle. The corpus callosum would be absent (Fig. 11a). In semi-lobar holoprosencephaly, the brain appears fused anteriorly and at the thalami with hypoplasia or agenesis of the corpus callosum (Fig. 11b). The mildest form of holoprosencephaly is the lobar type where subtle fusions of the frontal lobe and at the thalami is evident [86, 87]. The presence of frontal horns of the lateral ventricles and the extent of corpus callosum are valuable clues to differentiating semi-lobar from lobar holoprosencephaly with the lobar form of holoprosencephaly often demonstrating an element of frontal horn development as well as more advanced, if not complete, corpus callosum development. Several other entities have been described including septo-optic dysplasia and the middle interhemispheric variant of holoprosencephaly (Fig. 11c) [87–90]. Holoprosencephaly is often associated with midline craniofacial abnormalities, the severity of which often reflects the severity of the holoprosencephaly. It can also be syndromic with associated extra-CNS manifestations.

Holoprosencephaly results from disruption of the *SHH* pathway regulating the ventral induction of the prosencephalon. Hence, holoprosencephaly is also referred to as a “ventral induction failure” [89, 92]. Up to 50% of holoprosencephaly cases are linked to chromosomal number or structural abnormalities with trisomy 13, and less commonly trisomy 18, being the most common. Approximately 10% of the cases, on the other hand, are linked to CNVs [1, 91]. Finally, 18–25% are associated with SNV with *SHH*, *ZIC2*, *GLI2*, *SIX3*, *FGF8* and *FGFR1* being the most common holoprosencephaly genes [88, 91]. Accordingly, karyotyping, CMA and WES/WGS are all warranted as means of genetic investigations [1, 91]. A few genetic phenotype correlations have been established including the relation of certain holoprosencephaly genes (e.g.*ZIC2* and *SIX3*) to more severe forms of holoprosencephaly. Interestingly, though, there has been a wide variability in the severity of holoprosencephaly amongst family members with the same monogenic pathogenic variants. This suggests that the mode of inheritance is complex with variable expressivity. The effect of a given variant may also be modified by other genetic abnormalities or by environmental factors [91, 92]. 

## Dandy walker malformation

**Fig. 12:**
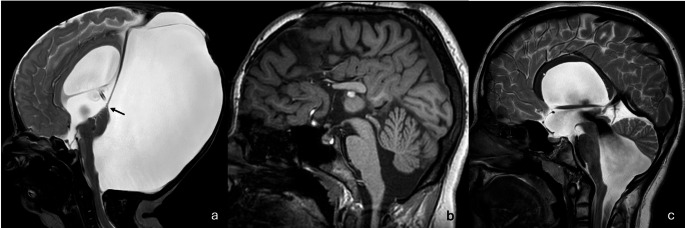
(**a**) Dandy Walker Malformation. Hypoplastic uprotated vermis forming the “tail” sign (arrow) associated with markedly enlarged posterior fossa communicating with the 4th ventricle, and supratentorial shunted hydrocephalus (**b**) Inferior Vermian Hypoplasia. Hypoplastic inferior vermis with preserved acute fastigial angle. Associated partial agenesis of corpus callosum (**c**) Blake’s Pouch Cyst/Diverticulum. Markedly widened fastigial angle and fourth ventricular outflow tract with slight supero-posterior displacement of the cerebellum and distortion of the inferior vermis

Dandy walker malformation (DWM) was historically characterized by (1) cerebellar vermian hypoplasia with cephalad rotation, (2) cystic dilatation of the fourth ventricle, and (3) a large posterior fossa with the torcula lying above the level of the lambdoid sutures (torcular-lambdoid inversion sign) (Fig. 12a). The last criterion, however, has been challenged as an enlarged posterior fossa is not exclusive to DWM but rather likely related to the severity of fourth ventricular obstruction [15]. Instead, new measures are added to the definition which include inferolateral displacement of the tela choroidea and choroid plexus [93], an obtuse fastigial recess and an enlarged tegmento-vermian angle. The criterion of vermian hypoplasia has also been more accurately described as “inferior-predominant” vermian hypoplasia with unpaired caudal lobules (Fig. 12a) [15]. Finally, the presence of “tail sign” which is primarily seen on fetal MRI can aid in confirming the diagnosis of DWM. The tail sign represents a dysmorphic thickened and elongated appearance of the nodulus along the inferior aspect of the vermis [94].

Correct identification of DWM is extremely important for prognostication and counseling purposes. DWM associated with other anomalies carry a worse prognosis when compared to isolated DWM and is correlated with the number and severity of concomitant intracranial and systemic anomalies [95]. Extensive literature discusses the differences between DWM and its mimics such as isolated inferior vermian hypoplasia (Fig. 12b) and Blake’s pouch cyst (Fig. 12c). These mimics were previously considered milder forms of DWM and labeled dandy walker “continuum” or “variants”, terms that are now discouraged given their propensity to result in misclassification as any non-DWM phenotypes were considered a Dandy Walker continuum. True DWM with normal posterior fossa was also inaccurately labeled as a continuum. Inferior vermian hypoplasia is distinguished from DWM by evaluation of the appearance of the fastigial angle which is obtuse in DWM while remains acute in inferior-vermian hypoplasia. Interestingly, this is attributed to the stage of cerebellar development at which malformation came about. In DWM, the timing of insult occurs earlier, hindering the internalization of the nodulus and so the fastigial stays obtuse. The timing of insult in vermian hypoplasia occurs later, after internalization of the nodulus and the reconfiguration of the fastigial recess into an acute angle (Fig. 12b) [15, 94, 96]. Blake’s pouch cyst results from failure of regression of the nonperforated embryonic Blake’s pouch at a later stage of posterior fossa development and results in secondary ventricular obstruction and a normal sized vermis. The differentiation of Blake’s pouch cyst and DWM may be at times challenging. In DWM, the tegmento-vermian angle is widened but can be variable in blake’s pouch cyst. Although the vermis is not hypoplastic in Blake’s pouch cyst, it may be distorted by the cyst giving false vermian measurements. At times, primarily on fetal MRI, the choroid plexus inferior to the vermis may falsely give the appearance of the tail sign. The location of the tela choroidea and choroid plexus has proven a valuable clue in such cases. In DWM, the tela choroidea and choroid plexus are displaced inferiorly and laterally while in Blake’s pouch cyst, it is displaced posteriorly under the vermis within the cyst (Fig. 12c).

The pathogenesis of DWM is unclear and involves both genetic and disruptive etiologies such as prenatal infection and it has also been associated with maternal diabetes and alcohol consumption. From a genetic stance, it is frequently linked to chromosomal abnormalities (e.g. trisomy 18) and CNVs, which therefore warrant cytogenetic testing [1, 15, 93, 95, 97, 98]. This can be performed prenatally for counseling purposes [97]. WES on the other hand has a diagnostic yield of only 16%[1] with only a few genes linked to DWM including *ZIC1*, *ZIC4* genes and *FOXC1*, all of which play a role in neurodevelopment [95]. 

## Joubert syndrome and related disorders

**Fig. 13:**
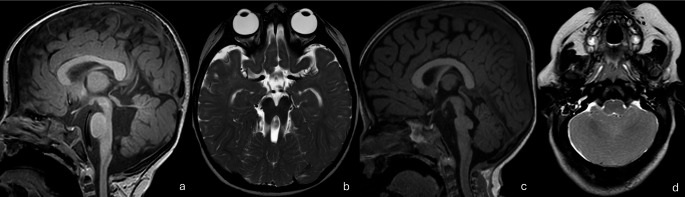
(**a**, **b**) Joubert syndrome. Thickened, horizontally oriented superior cerebellar peduncles and vermian hypoplasia (A), along with a deep interpeduncular fossa producing the classic “molar tooth sign” on axial MRI (B) (**c**) Tegmental cap dysplasia. Dorsal “cap-like” bulge of the pontine tegmentum, and dysplastic brainstem, and vermo-cerebellar hypoplasia/dysplasia (**d**) Rhombencephalosynapsis*.* Agenesis of the cerebellar vermis with fusion of the cerebellar hemispheres and dentate nuclei, resulting in a characteristic midline “fused cerebellum” appearance

Joubert syndrome (JS) and related disorders are CNS ciliopathies with a wide clinical spectrum. JS is a form of midbrain-hindbrain junction malformation and radiologically demonstrates cerebellar hypoplasia with a dysplastic vermis, elongated horizontal superior cerebellar peduncles and deep interpeduncular fossa giving the well-described “molar tooth” sign (Fig. 13a, b)[1, 100–102]. They are divided into several subtypes including Classic JS, JS with retinal disease, JS with renal disease, JS with oculo-renal disease, JS with hepatic disease, JS with oro-facial-digital features, JS with acrocallosal features and JS with Jeune asphyxiating dystrophy [99, 100]. Genetic mutations are found in more than 35 genes. In familial JS, 40–75% of cases are related to specific mutations in *AHI1*,* CPLANE1*,* CC2D2A*,* CEP290*,* KIAA0586*,* MKS1 and TMEM67*. Therefore, ethnicity-adapted gene panels can be used as a first-line test. Otherwise, WES or WGS are preferred and offer higher sensitivity [100, 102]. Cytogenetic analysis is generally not warranted, with one exception: JS in the setting of nephronophthisis where 25% of cases have a *NPHP1* CNV, and accordingly, CMA is recommended [1].

## Rhombencephalosynapsis

Rhombencephalosynapsis is defined as the complete, or rarely partial, fusion of the cerebellum, dentate nuclei and superior cerebellar peduncles (Fig. 13c). It is rarely an isolated anomaly and is often described in syndromic contexts, in association with other CNS or extra CNS anomalies [1, 33]. Rhombencephalosynapsis has been divided into four subsets, (1) rhombencephalosynapsis associated with Gómez-López-Hernández syndrome; (2) rhombencephalosynapsis with VACTERL association (Vertebral defects, Anal atresia, Cardiac defects, Tracheo-Esophageal fistula, Renal anomalies, and Limb abnormalities); (3) rhombencephalosynapsis with atypical holoprosencephaly, and (4) isolated rhombencephalosynapsis. Gómez-López-Hernández syndrome is a phakomatosis in which patients typically present with parieto-occipital alopecia, facial dysmorphism and trigeminal anesthesia. Common CNS associations include mesencephalosynapsis, aqueductal obstruction and secondary hydrocephalus, corpus callosum dysgenesis, absent septum pellucidum, fused fornix and fused thalami which suggest a common developmental etiology with holoprosencephaly. Chromosomal abnormalities have been identified in patients with rhombencephalosynapsis and therefore cytogenetic analysis is often performed [103]. WES/WGS, on the other hand, are not routinely indicated, unless other anomalies are present that warrant further testing (e.g., holoprosencephaly).

## Genetically Unresolved Malformations

Several anomalies such as pontine tegmental cap dysplasia (Fig. 13c), and diencephalon-mesencephalon junction dysplasia are diagnosed radiologically without clear genetic abnormalities identifiable on genetic investigations at this time [1, 33]. 

## Conclusion

In the era of precision medicine, pediatric neuroradiologists are expected to understand not only the imaging features of CNS pathologies but also their genotypic correlations, as this can greatly impact clinical management and treatment development. Exploring the pathogenesis of brain malformation has proven to be challenging due to the complex nature of brain development. In addition, the influence of disease mosaicism, incomplete penetrance and variable expressivity in brain malformation have created hurdles in recognizing the underlying genetic culprits. Fortunately, advancements in genetic testing and the promotion of epilepsy surgery have significantly improved diagnostic yield. There has also been a plethora of studies examining the relevant pathways (e.g., MAP kinase pathway), cellular structures (e.g. tubulin cytoskeleton) and embryology that have significantly improved our understanding of brain malformation. This review aims to establish a foundational understanding of brain malformations in relation to embryology and genetics. Given the breadth and complexity of the topic, this paper does not attempt to provide an exhaustive account but rather highlights key concepts most relevant to clinical practice.

## Data Availability

No datasets were generated or analysed during the current study.
